# Diagnostic Significance of Intracystic Nodules on MRI in Rathke's Cleft Cyst

**DOI:** 10.1155/2012/958732

**Published:** 2012-09-12

**Authors:** Shou-sen Wang, De-yong Xiao, Ying-hao Yu, Jun-jie Jing, Lin Zhao, Ru-mi Wang

**Affiliations:** ^1^Department of Neurosurgery, Fuzhou General Hospital, Fuzong Clinical College, Fujian Medical University, 156 Xihuanbei Road, Fuzhou 350025, China; ^2^Department of Pathology, Fuzhou General Hospital, Fuzong Clinical College, Fujian Medical University, 156 Xihuanbei Road, Fuzhou 350025, China

## Abstract

*Background and Purpose*. To explore strategies for the diagnosis and treatment of Rathke's cleft cyst (RCC). *Methods*. The medical records of 24 patients with sellar RCC were retrospectively reviewed. Two patients had concomitant pituitary adenoma, 2 underwent transcranial surgery, and 22 underwent transsphenoidal surgery. The clinical features, especially the findings of intracystic nodules on MRI, were evaluated and compared with the pathological findings. *Results*. Preoperatively, only 2 patients were diagnosed with RCC or suspected RCC. Pre- and postoperative MRI images revealed 10 intracystic nodules in 9 (37.5%) patients. Two nodules had bull's eyelike changes. The signal intensity of the intracystic nodules varied on T1- and T2-weighted images. Not all nodules on T2-weighted images were visualized. Postoperative MRI revealed recurrence or residual lesion in 5 patients; none had new symptoms and a second surgery was not required. *Conclusions*. Identifying intracystic nodules is important in patients with sellar cystic lesions. Bull's eyelike change in an intracystic nodule on MRI, which is reported here for the first time, potentially might have value for confirming the diagnosis.

## 1. Introduction

Rathke's cleft cyst (RCC) is a benign, epithelium-lined intrasellar cyst. It is believed to originate from remnants of Rathke's pouch, and its initial site is located in the middle lobe of the pituitary gland. The clinical manifestations of RCC are characterized by headache, hypopituitarism, visual disorder, and diabetes insipidus. Imaging examinations usually indicate a lesion located in the sellar or suprasellar region. Rathke's cleft cyst is found in 12–33% of routine autopsies [1, 2]. Some RCCs grow progressively over time [1, 3], but few patients with RCCs have obvious clinical symptoms. However, rupture of a RCC may cause severe symptoms [4–7], and RCC is prone to recurrence to a certain extent following surgery [8]. In some patients, RCCs remain stable during long-term followup, whereas some RCCs may undergo reduction after treatment with glucocorticoid [9]. In several cases, RCCs have resolved spontaneously [10]. 

Although several clinical studies on RCC have been conducted, the precise source and natural history of RCC are still poorly understood. The features of RCCs on imaging are still not clearly defined, and thus many patients are misdiagnosed before surgery. In addition, indications for surgery are controversial, as are specific operative procedures that are used. Even with modern imaging techniques, it can be difficult to distinguish RCCs from other types of cystic sellar lesions such as cystic pituitary adenoma and craniopharyngioma [11]. It may be somewhat easier to differentiate RCCs from craniopharyngiomas than from intracellular arachnoid cysts because craniopharyngiomas are more associated with psychiatric symptoms and calcifications or solid components on imaging [12]. It has also been reported that single-shot fast spin-echo diffusion-weighted MR imaging is of value for differentiating RCCs from craniopharyngiomas and hemorrhagic components of pituitary adenomas, but not from cystic components of pituitary adenoma [13]. Making a preoperative diagnosis is important because various types of cystic sellar lesions are treated differently. Often the minimally invasive transsphenoidal endoscopic approach is used for symptomatic RCC. 

Obtaining an accurate preoperative diagnosis, determining the proper surgical indications, and selecting the optimal surgical methods continue to be the major concerns of neurosurgeons. In the present study, the medical records of 24 patients with sellar RCC in the past 7 years were retrospectively reviewed, and the findings on imaging and pathological examinations were compared with the aim of proposing improved diagnostic approach and treatment strategy for RCC. 

## 2. Methods 

### 2.1. General Information

A total of 24 patients (6 male and 18 female) with RCC treated at our hospital from January 2005 to August 2011 were included. The average age was 40 years (range: 20–62 years). Patients were excluded if they underwent surgery not performed by the first author or received conservative therapy. All patients were followed up by face-to-face interview or telephone. The follow-up period ranged from 4 weeks to 6.5 years (median of 25 months). 

### 2.2. Imaging Examinations

MRI was performed on all patients utilizing the following 2 systems and parameters: (1) GE Signa 1.5T MR system—routine MRI head coil using fast spin-echo (FSE) or spin-echo (SE) pulse sequences. Coronal and sagittal T1-weighted images and coronal and axial T2-weighted images were obtained. At the same time, enhanced three-dimensional scanning was also performed using GD-DTPA (0.1 mmol/kg) as the contrast agent. For coronal and sagittal images, slice thickness: 3 mm, slice interval: 3 mm; for axial images, slice thickness: 6 mm, slice interval: 7.5 mm; Parameters: T1WI: tr500 ms, TE15 ms, matrix: 320 × 192, field of view: 220 × 220 mm; T2WI: TR4200 ms, TE98 ms, matrix: 384 × 256, field of view: 192 × 240 mm, 2 excitations. (2) Siemens Magnetom Trio Tim 3.0 T MRI system—axial thickness: 5 mm; slice interval: 6.5 mm; coronal and sagittal thickness: 2.5 mm; slice interval: 2.75 mm. Parameters: T2WI: coronal and axial images: TR/TE 3000 ms/83–98 ms; field of view: 180 × 180 mm; matrix: 384 × 252; 2 excitations. T1WI: coronal and axial images: TR/TE 400–500 ms/8 ms; field of view: 180 × 180 mm; matrix: 320 × 240; 3 excitations. Enhanced three-dimensional scanning: contrast agent: GD-DTPA at 0.1 mmol/kg. 

### 2.3. Surgical Methods

All patients underwent surgery due to mass effect of a space-occupying lesion. Additional reasons for performing surgery were headache that could not be explained by other etiologies, decreased visual acuity or increased visual field defect, and endocrine dysfunction. Craniotomy was performed via the pterion approach in 2 patients, and via the unilateral nasal-nasal septum-sphenoid sinus (transsphenoidal) approach in the remaining 22 patients. In transcranial surgery, the RCC contents were suctioned and most or all of the cyst wall was resected. In transsphenoidal surgery, following incision of sellar dura mater, RCC was located beneath the dura mater, and the cystic fluid was removed by suction before resecting the cyst wall. In cases where normal pituitary gland was located beneath the dura mater, the RCC was usually light yellow, yellow, or grey-white and could be either softened or hardened. The pituitary gland would be incised and the lesion removed after the cystic region was reached. The tissues surrounding the cyst were collected for pathological examination. After surgery, the sellar floor was opened to assure smooth drainage.

### 2.4. Endocrine Examination

Preoperative and postoperative endocrinological lab tests were conducted, including tests for adrenocorticotropic hormone (ACTH), growth hormone (GH), thyroid-stimulating hormone (TSH), testosterone, cortisol, estradiol, prolactin, and follicle-stimulating hormone/luteinizing hormone (FSH/LH).

### 2.5. Histopathological Examination

Surgical specimens were diagnosed as RCC via routine pathological examination. Tissues were routinely embedded in paraffin and cut into sections which were stained with hematoxylin and eosin (H&E). 

## 3. Results

Only 2 of the 24 patients were diagnosed with RCC or suspected RCC before surgery, and 2 were diagnosed with sellar cystic occupying lesions. In the remaining patients, tumor or pituitary adenoma was suspected in the preoperative diagnosis. 

### 3.1. Imaging Findings

**Figure 1:**
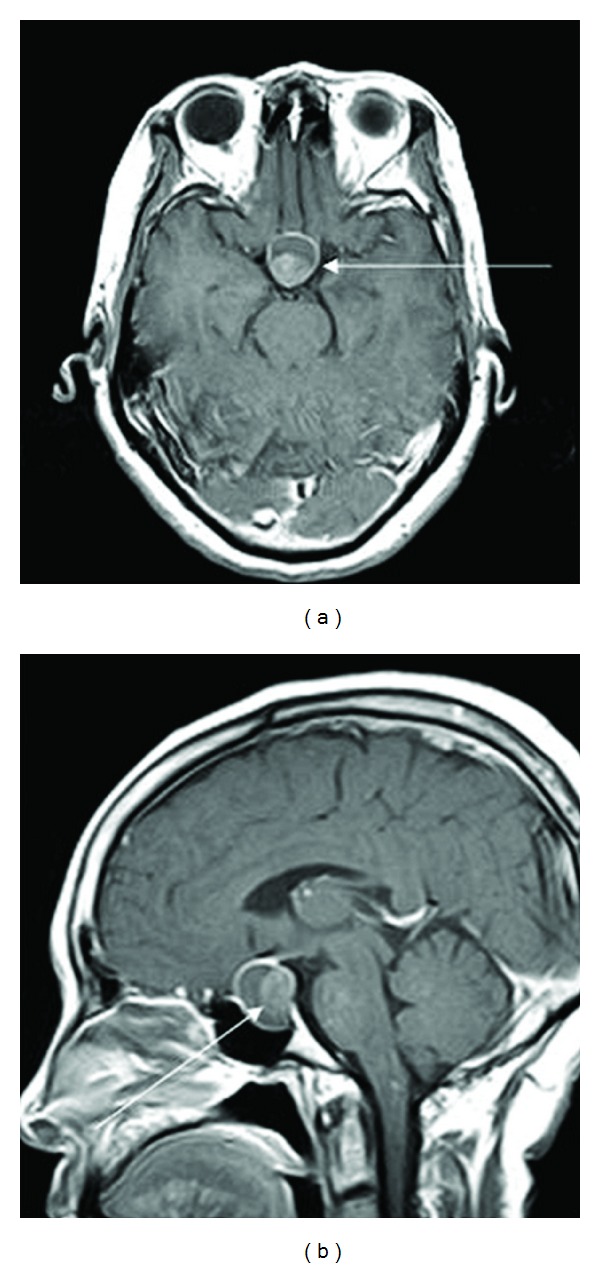
Case 2: enhanced MRI T1-weighted images. Intracystic nodules with hyperintense signal on T1-weighted images in the posterior RCC (arrow).

**Figure 2:**
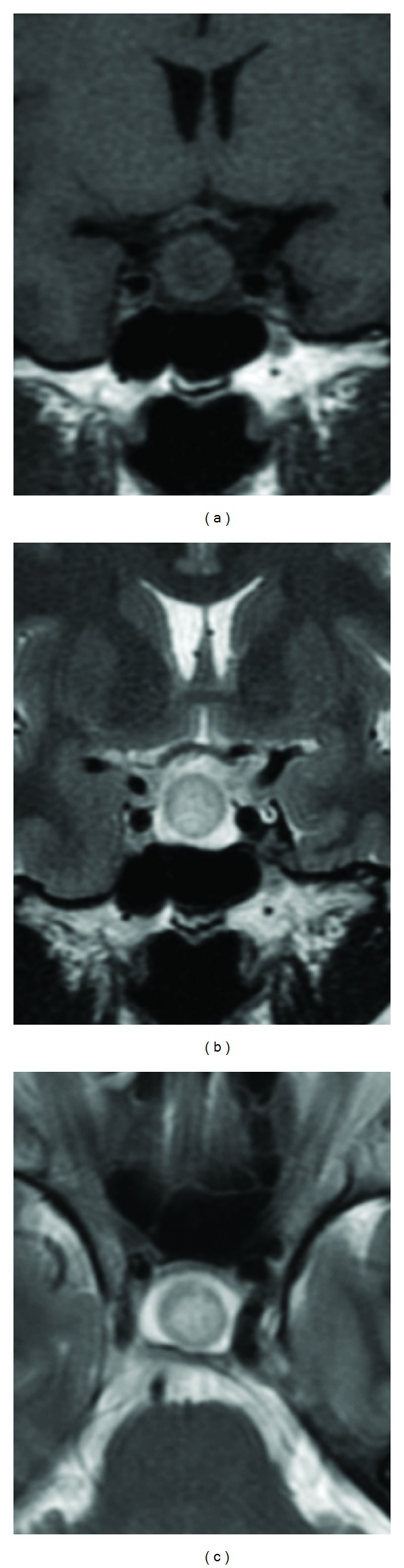
Case  10: nonenhanced MRI shows bull's eyelike intracystic nodules in RCC. Cyst fluid with hypointense signal in T1-weighted images and hyperintense signal in T2-weighted images. Circular isointense signal in T1- and T2-weighted images surrounding the nodule, and slight hypointense signal in T1-weighted image and slight hyperintense signal in T2-weighted images at the center of cyst. (a) nonenhanced coronal T1-weighted image; (b) coronal T2-weighted image; (c) axial T2-weighted image.

**Figure 3:**
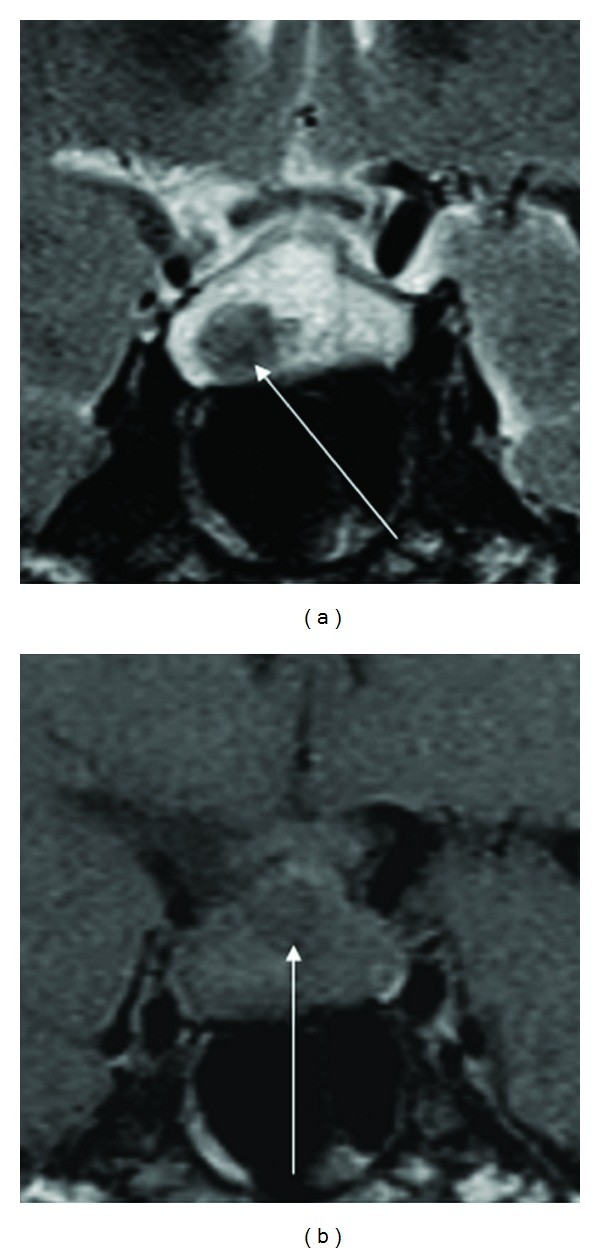
Case 17: nonenhanced coronal MRI images of RCC. (a) T2-weighted image: nodule with hypointense signal at right lower part (arrow). (b) T1-weighted image: nodule with hypointense signal at the upper part (arrow).

**Figure 4:**
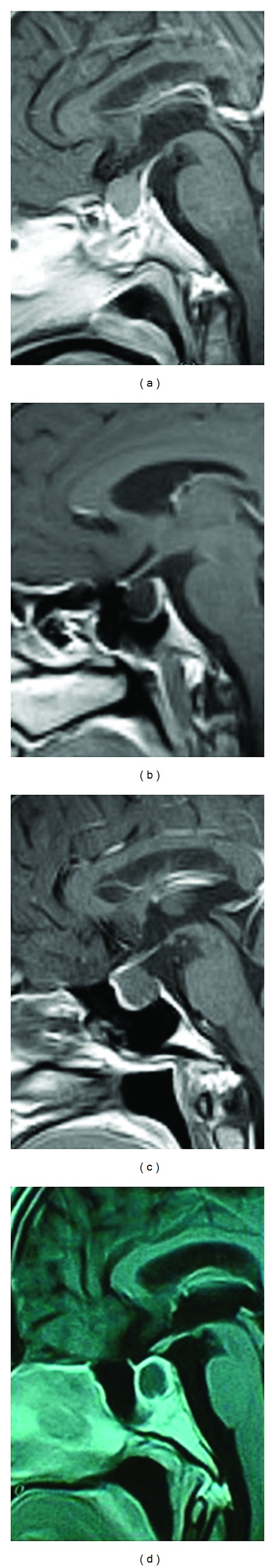
Enhanced middle sagittal image: relation between RCC and adenohypophysis at 4 different sites. (a) RCC anterosuperior to adenohypophysis. (b) RCC anterior to adenohypophysis. (c) RCC posterior to adenohypophysis. (d) RCC surrounded by adenohypophysis.

The imaging findings are presented in Table 1. According to the images and surgical findings, the mean maximal cyst size was 14.5 mm in diameter (range: 7.0–30.0 mm). Cyst size appeared to be related to blurry vision; for lesion size > 15 mm, there was a greater incidence of decreased visual acuity and increased visual field defect, and for cyst size ≤ 15 mm, visual acuity appeared to be unaffected but visual field defect was noted without central involvement. A slightly lateral location was observed for the RCC in 2 patients and in the remaining patients, RCCs were located in the midline of the pituitary fossa. Evident intracystic nodules were found in 8 patients (Figures 1, 2, and 3) and a suspected nodule in one patient. In all, 10 intracystic nodules were identified by MRI in 9 patients, and the nodules had homogenous intensity without enhancement. One patient (case 17) had 2 nodules within the RCC. Two nodules had bull's eyelike changes (cases 10 and 19) (Table 2). In the 9 patients with intracystic nodules, puncturing of the sellar dura mater during transsphenoidal surgery led to the flow of thin mucous fluid. After opening the sellar floor and pituitary tissue layer, both thin and thick mucous fluid, and semisolid or solid jellylike tissues were present, but blood supply was absent. Because the thin fluid flowed out immediately during the operation, the tissues could not be comprehensively observed. Thus, the location of intracystic nodules could not be accurately verified during surgery. Observations of sagittal MRI images revealed a cyst anterior to the adenohypophysis in one patient, anterosuperior to the adenohypophysis in 2, posterior to the adenohypophysis in 15, surrounded by the pituitary in 4 patients (Figure 4) and located between the pituitary and tumor in 2 patients. Morphologically, the majority of cysts were round or oval, and the minorities were snowman-like, spindle-like, pancake-shaped or boot-shaped (Table 1). In several patients, the cysts presented characteristic waists. 

In all patients, the postoperative diagnosis of RCC was based on the intraoperative findings. In 2 patients with concomitant pituitary adenoma, resection of the pituitary adenoma was performed. In case 7, the RCC was located at the apex of the tumor cavity, which was separated by a thin and uneven transparent membrane; the RCC was not removed directly. In case  24, both the RCC and pituitary adenoma were completely removed. In the remaining patients, the intracystic contents were completely removed. 

After surgery, transient diabetes insipidus was present in about 50% of patients and it continued for 3 days to 2 weeks. Diabetes insipidus in 3 patients that lasted for one month resolved spontaneously. In case 13, the patient already had diabetes insipidus for several years and postoperative urinary volume returned to the normal level. In the 8 patients with visual impairment before surgery, eyesight was improved after surgery. Postoperative visual acuity improved by a mean of 0.35 (range: 0.1–0.6). Among 17 patients with pre-operative headache, 8 had improvement and only had mild headache postoperatively; among 9 patients who had pre-operative dizziness, 4 had improvement and only had mild dizziness postoperatively. 

 Following surgery, patients were reexamined with MRI and no recurrence was noted in 21 of them. 

## 4. Discussion

### 4.1. New Features of Intracystic Nodules of RCC and Their Significance

In the present study, the MRI findings of 24 patients were reviewed and 9 patients (37.5%) had intracystic nodules. Most of the nodules had homogenous intensity without enhancement on MRI. Intracystic nodules have been investigated in several studies and are regarded to have diagnostic value [10, 11, 14]. We found that 2 intracystic nodules were characterized by bull's eyelike changes. This is the first time that bull's eye-like changes are reported in sellar intracystic nodules. The bull's eye-like changes might be specific for RCC and provide valuable information for confirming the diagnosis, although it cannot be ruled out that these changes might also occur in other sellar masses. Further research is needed to determine whether the bull's eye-like changes are specific to RCCs. The mechanism underlying the formation of bull's eye-like changes is still unclear, and further research is required for understanding how these changes occur. 

Several nodules were only clearly present on T1-weighted images and 2 nodules with different intensities were found within a single cyst. These features have also never been reported before. In our study, we also observed that the intensity of intracystic nodules varied on T1- and T2-weighted images. These cysts on T1-weighted images could have lower intensity than cystic fluid, and not all cysts could be visualized on T2-weighted images. These findings are not consistent with previous reports, which found that the cysts were hyperintense on T1-weighted images and hypointense on T2-weighted images, and always could be clearly identified on T2-weighted images [10, 11, 14]. 

A minor fraction of cysts can demonstrate ring enhancement. Billeci et al. [1] observed ring-enhancement in 2 of 14 RCC patients. In our study, 4 patients had ring enhancement (cases 4, 16, 17, and 19). Some researchers have proposed that ring enhancement on MRI was related to squamous metaplasia, inflammation, and deposition of hemosiderin or cholesterol crystals [15]. Other researchers have also speculated that this enhancement was caused by the thin pituitary tissues surrounding the RCC [1, 16], which is consistent with our findings. Of note, pituitary abscess and RCC have extremely similar ring enhancement and patients with these lesions also present with fever and inflammatory response, and have abscess-like cystic fluid. However, inflammatory granulation tissue should surround the cystic fluid in pituitary abscess, which may also demonstrate changes related to sphenoid sinusitis on imaging examinations.

In some studies, researchers have speculated that the cystic fluid of RCC was mainly composed of proteins and cholesterol, although the cholesterol content was extremely low [3, 10, 16]. In the present study, MRI revealed that the intensity of intracystic nodules varied. The viscosity and texture of intracystic contents also varied as demonstrated by the surgical findings. Hematoxylin and eosin staining of RCC samples revealed that the cystic fluid contained proteins of different sizes and had a red/blue staining pattern. These findings imply that the appearance of intracystic nodules may be related to the concentration of mucins and have multiple proteins. 

Nishioka et al. [14] reported that intracystic nodules were identified in 16 of 37 patients (43.2%), Byun et al. [10] identified intracystic nodules in 10 of 13 patients (76.9%), and Binning et al. [11] observed intracystic nodules in 9 of 20 patients (45.0%). The incidence of intracystic nodules was relatively high in these studies. In our study, while a lower incidence of 37.5% is reported, it is worth noting that intracystic nodules were not even observed in some studies [1, 15–17]. Intracystic nodules with nonenhancement and homogenous intensity could be used to differentiate RCC from other cystic lesions in the sellar region. Therefore, more attention should be paid to the presence of intracystic nodules in clinical practice. 

Distinguishing among RCC, craniopharyngioma, and cystic lesions remains a challenging diagnostic problem [12]. To address this issue, Shin et al. [12] used clinical, biochemical, and radiographic features to identify distinctive characteristics. Patients with craniopharyngioma had significantly more psychiatric symptoms than patients with RCC or arachnoid cyst, and significantly fewer patients with arachnoid cyst had two or more impaired pituitary axes than patients with RCC or craniopharyngioma. On CT scan, a significantly higher percentage of patients with craniopharyngioma had calcification detected than patients with RCC or arachnoid cyst.

### 4.2. Determination of Surgical Indications Based on the Therapeutic Efficacy

The goal of surgical intervention is to remove the intracystic contents and eliminate the source of space-occupying lesions and pituitary inflammation. The cyst wall is also partially resected for pathological examination for diagnostic confirmation. Opening of the sellar floor is beneficial for the drainage of cystic fluid. Some researchers have proposed that early surgical intervention is necessary for RCC patients in order to prevent the aggravation of pituitary function [1, 18]. The inflammatory response in the pituitary tissues surrounding the cyst is not found in pituitary adenoma and has become an intrinsic indicator for active surgical intervention in symptomatic RCC. Other researchers have recommended that surgery is required even when the symptoms are mild [1]. Of note, protecting endocrine function should be more important than resection of the RCC. Attempting to remove as much cyst wall as possible may compromise pituitary function and even worsen diabetes insipidus. We believe that while the intracystic contents should be completely removed, biopsy or partial resection of the cyst wall should also be simultaneously performed. Aggressive resection of the cyst wall is not recommended, and this has also been noted in other studies [19]. We would rather perform surgery following recurrence, instead of performing radical resection of the cyst wall that may lead to compromised endocrine function. For patients with recurrent RCC, the indications of surgery should be based on the clinical manifestations (especially new symptoms). Mere findings on MRI are not indications for surgery. In the present study, patients with recurrence did not undergo a second surgery because the growth of recurrent RCC stopped without developing new symptoms. Finding typical cystic contents during surgery is helpful for the surgical diagnosis, and observing typical cystic epithelial cells on histological examination is not necessary for diagnostic confirmation. It is important to note that surgery is associated with risk of damaging the pituitary tissues, especially when the RCC is located behind the adenohypophysis. 

This study had several limitations. First, it is a retrospective case series. Second, the sample size of 24 patients was small. Third, the length of followup, which ranged from 4 weeks to 6.5 years, was less than optimal. Finally, it can be extremely difficult, if not impossible, to distinguish RCC from cystic craniopharyngioma in many cases unless sufficient lesional epithelium is removed for detailed histologic examination. In 6 patients (cases 1, 4, 11, 12, 15, and 16), adequate specimen for detailed histologic examination was not obtained.

## 5. Conclusions

We made several unique observations. The most notable was that intracystic nodules in RCC can have bull's eye-like changes; these changes might be specific to RCC. In addition, 2 nodules with different intensities were found within a single RCC. Also, we found that, for the first time, that some nodules are only clearly present on T1-weighted images, while being difficult to distinguish on T2-weighted images. These findings should be helpful in diagnosing RCC preoperatively, which is an important goal because different types of sellar cysts require different forms of treatment.

## Figures and Tables

**Table 1:** Imaging findings for patients with Rathke's cleft cyst.

Case	Age/gender	Symptoms	Hormonal profile	Imaging findings		
				Height of lesions (mm)	MRI signals	Direction of migration
1	50/M	Headache	Normal	13	High T1 and T2	Posterior
2	59/F	Headache, dizziness, blurry vision	Reduced cortisol	22	Low T1, high T2	Posterior
3	59/M	Headache	Reduced estradiol	19	High T1 and T2	Posterior
4	30/M	Headache, blurry vision	Normal	17	Low T1, high T2	Surrounded by pituitary
5	62/F	Blurry vision, loss of consciousness	Reduced prolactin	15	Slightly high T1, low T2	Anterosuperior
6	35/M	Headache	Elevated cortisol and testosterone	15	Low T1, high T2	Posterior
7	22/F	Headache, blurry vision	Elevated prolactin and growth hormone	13 mm (anteroposterior)	High T1 and T2	Cyst located between pituitary and tumor
8	41/F	Galactorrhea, abnormal menstruation	Elevated prolactin and thyroid-stimulating hormone	9	Isointense T1, high T2	Posterior
9	60/F	Dizziness, blurry vision, weakness in extremities	Reduced estradiol	13	Slightly low T1, high T2	Posterior
10	54/F	Headache, dizziness	Reduced estradiol	15	Low T1, high T2	Posterior
11	32/M	Blurry vision	Reduced FSH, LH, and elevated cortisol	13.5	Slightly low T1, Isointense T2	Anterosuperior
12	41/F	Headache, dizziness, abnormal menstruation	Normal	15	High T1 and T2	Posterior
13	28/F	Headache	Reduced FSH and LH	11	Slightly high T1, high T2	Posterior
14	42/F	Headache, polyuria, polydipsia	Elevated estradiol	14	High T1 and T2	Posterior
15	48/F	Headache, dizziness	Elevated T4. Reduced cortisol and estradiol	14	Low T1, high T2	Anterior
16	20/F	Headache	Normal	10	Slightly low T1, high T2	Surrounded by pituitary
17	29/F	Headache, dizziness	Normal	11	Isointense T1, high T2	Surrounded by pituitary
18	46/F	Headache, dizziness, blurry vision	Elevated ACTH, T3, FT3. Reduced TSH, and testosterone	26	Slightly high T1 and T2	Posterior
19	40/F	Headache, abnormal menstruation	Normal	14.6	Isointense T1, slightly high T2	Surrounded by pituitary
20	20/M	Headache, dizziness	Elevated estradiol	7	Isointense T1, high T2	Posterior
21	37/F	Headache, dizziness, abnormal menstruation	Normal	20	Isointense T1, high T2	Posterior
22	54/F	Dizziness, generalized weakness	Reduced estradiol	7.5	High T1, isointense T2	Posterior
23	31/F	Headache	Reduced cortisol	18	Isointense T1, high T2	Posterior
24	34/F	Blurry vision, amenorrhea, galactorrhea	Elevated prolactin. Reduced estradiol	15	High T1, isointense T2	Cyst located between pituitary and tumor

**Table 2:** Features of intracystic nodules on MRI in RCC patients.

Case	Intensity of nodules	Cyst diameter/nodule diameter (mm)	Location and shape of nodules
2	Homogenously hyperintense in T1	22.0/17.0	Oval
9	Hyperintense in T1	13.0/10.0	Ball-like
10	At the periphery, isointense in T1, isointense ring in T2; in the center, slightly hypointense in center on T1, slightly hyperintense in T2 with Bull's eyelike changes	15.0/7.0	Ball-like
12	Isointense in T2, slightly hyperintense in T2 with more homogenous distribution	13.5/10.0	Ball-like
13	Hyperintense in T1, slightly hypointense in T2	11.0/7.9	Triangle at sagittal images, similar to the outline of cyst in morphology
17	Two nodules with homogenous intensity: (1) presence in T2, slightly hypointense in T2; (2) presence in T1, slightly hypointense in T1	11.0/7.011.0/6.3	In the right lower and upper central part, ball-like
18	Isointense in T1, isointense in T2	26.0/14.2	In the lower part, ball-like
19	At the periphery, slightly hyperintense in T1, slightly hyperintense ring in T2; in the center, isointense in T1, slightly hypointense in T2, suspected Bull's eyelike changes	14.6/4.1	In the right lower part, ball-like
23	Hyperintense in T1, hypointense in T2	18.0/8.0	In the lower and middle part. Ball-like, atypical nodules
